# Expecting the unexpected: Examining the interplay between real-world knowledge and contextual cues during language comprehension

**DOI:** 10.3758/s13421-025-01689-x

**Published:** 2025-02-06

**Authors:** Chengjie Jiang, Ruth Filik

**Affiliations:** School of Psychology, University of Nottingham, University Park, Nottingham, NG7 2RD UK

**Keywords:** Language comprehension, Context, World knowledge, Sentence completion, Self-paced reading

## Abstract

Communication frequently involves discussions about real-world implausible events. Since most prior research used specific contextual cues to indicate a strong bias towards real-world knowledge violations, it remains unclear how real-world and contextual knowledge interact when the context is relatively unconstraining (e.g., dream scenarios), where both plausible and implausible information is supported. We investigated this issue using sentence completion (Experiment 1) and self-paced reading tasks (Experiment 2). Results of Experiment 1 showed that comprehenders were guided by the dream context to expect less plausible information in a general way, but their expectations were still largely constrained by real-world knowledge. Results of Experiment 2 showed that although comprehension in such contexts was initially guided by real-world knowledge, plausible information became more difficult to comprehend than implausible information (e.g., “putting meat and vegetables in the refrigerator_plausible_/wardrobe_implausible_”) at the final regions of the target sentence. Our study is the first to show that context is powerful enough to guide comprehenders towards expecting world knowledge violations even without explicit constraints indicating this bias, which is mainly driven by increased comprehension difficulties for plausible contents rather than decreased difficulties for implausible ones. Importantly, our findings raise new questions about how comprehenders switch from an old situation model to a new one. They also indicate necessary extensions for language comprehension models, highlighting that information unrelated to both real-world and contextual knowledge in any direct way (i.e., information with extremely low cloze probability) can still be ultimately preferred in certain contexts.

## Introduction

Human communication frequently entails discussions about possible worlds with alternative realities. In daily conversations, people often talk about counterfactual scenarios (how things could have been different) as well as fictional worlds depicted in novels, films, TV series, video games, etc. In fact, discussing events that are not temporally or spatially present is a significant feature of human language, as it is arguably what distinguishes human language from the communication systems of most other animals. This cognitive process becomes most profoundly observable when the events being discussed are so different from reality that they are unlikely to ever occur. However, although fictional and counterfactual contexts can facilitate discussions of real-world implausible events, world knowledge still has an undeniable influence on language processing. Consequently, the comprehension of these implausible events is unlikely to be cost-free. Thus, it is particularly important to understand how real-world and contextual knowledge interact during language comprehension.

### The influence of contextual constraints on the world knowledge effect

Previous studies have demonstrated that world knowledge violations cause disruptions in comprehension (e.g., Cook & Guéraud, 2005; Cook & Myers, 2004; Cook et al., 2018; Hagoort et al., 2004; Kuperberg & Jaeger, 2015; Rayner et al., 2004; Williams et al., 2018), indexed by longer reading times in self-paced reading and eye-tracking experiments, as well as larger N400 amplitudes in event-related potential (ERP) experiments, that is, the world knowledge effect. However, in some cases, contextual modulation seems to attenuate or even override the world knowledge effect, in that discourse contexts can adjust what is perceived by comprehenders as plausible. For example, context can facilitate the processing of real-world implausible information by activating one’s knowledge of a previously known fictional world (e.g., the Incredible Hulk; Creer et al., 2018; Filik, 2008; Filik & Leuthold, 2008, 2013; Foy & Gerrig, 2014; Saerys-Foy et al., 2022; Troyer & Kutas, 2018; Troyer et al., 2020). Furthermore, contexts can even create a new “unrealistic world” (e.g., a fictional or counterfactual world) that contradicts well-known historical events (e.g., a world where “NASA did not develop its Apollo Project”; Nieuwland & Martin, 2012) or biological and physical laws (e.g., a world where “a lion could speak”; Creer et al., 2018; Ferguson & Sanford, 2008; Ferguson et al., 2008; Nieuwland, 2013; Nieuwland & Van Berkum, 2006; Saerys-Foy et al., 2022; Soares et al., 2023; Walsh et al., 2018). In such cases, comprehenders’ knowledge of the contextual world has an impact on language comprehension that is equally strong, or sometimes even stronger, than their knowledge of the real world. As a result, as long as real-world implausible information is consistent with the contextual world, it does not induce increased reading times or N400 amplitudes compared with real-world plausible information. Hence, the contextual modulation can lead to an attenuated, or even reversed, world knowledge effect, that is, the contextual effect. This effect occurs even when only a minimal unrealistic description is provided (Soares et al., 2023).

Of note, most of these existing studies have adopted contextual cues biased towards world knowledge violations, such as a detailed set-up story (e.g., Creer et al., 2018; Nieuwland & Van Berkum, 2006) or at least a minimal fictional description (Soares et al., 2023), to license real-world implausible information. In other words, in these studies, it was clearly specified in what exact way the unrealistic contextual world was different from the real world, and naturally, comprehension was constrained by those rules. For example, in a context that portrayed a peanut singing about a girl he had met, it was explicitly indicated that the peanut behaved in the same way as humans and no longer resembled food. As a result, in such a scenario, the peanut engaging in food-related actions, such as being seasoned with salt, severely violated these contextual constraints, leading to comprehension difficulties. In contrast, the peanut engaging in human-like actions, such as exhibiting affection, though real-world implausible, was strongly supported by the context and thus could be anticipated by comprehenders (Nieuwland & Van Berkum, 2006). Therefore, the reduced processing difficulties for real-world implausible information observed in these contexts were primarily due to the explicit and specific contextual support provided for such information.

In fact, it has been found that the strength and timing of the contextual effect is modulated by the amount of related contextual cues that had been built up in the discourse. For example, through four self-paced reading experiments, Walsh and colleagues (2018) found that even with abundant contextual support for real-world implausible events, such events still caused processing difficulties when they were unrelated to the fantasy context. Furthermore, when real-world implausible events were related to the fantasy context, if they were inconsistent with prior context at the local level, they caused immediate disruption; if they were inconsistent with prior context at the global level, disruption was initially eliminated but still observed later on.

Following these findings, it seems that the contextual effect may only be observable when real-world implausible information is supported by certain contextual constraints. But is this really the case? Empirical evidence is divided on this issue. For example, in an eye-tracking experiment conducted by Cook and Myers (Experiment 2, 2004), participants read a weakly constraining context (e.g., a movie was being filmed, but due to a low budget and small staff, everyone had to take on extra jobs and responsibilities) followed by information that was either real-world plausible or implausible (e.g., the script was rehearsed by the actress_plausible_ vs. “Action!” was called by the actress_implausible_). Despite contextual support for world knowledge violations, there was no specific support for the target implausible event. As a result, although the world knowledge effect was absent in the early stages of comprehension (first-pass reading times in the target region), it became observable in subsequent measures (second-pass reading times in the target region and first-pass reading times in the post-target region).

However, other studies have shown contrasting findings (Filik & Leuthold, 2013; Warren et al., 2008). For example, Warren and colleagues (2008) conducted an eye-tracking study in which participants read a context about a famous fictional character (e.g., Harry Potter) doing something either real-world plausible or implausible (e.g., “used a microwave to heat_plausible_/teach_implausible_ the tough bread”). In the fictional context, the world knowledge effect was only observed in early (first fixation and gaze duration on both the target and spillover regions), but not late (go-past and total reading times on both the target and spillover regions), stages of comprehension. Interestingly, “teach the bread” was not only inconsistent with real-world knowledge, but also unsupported by context (since there was no indication in any of the books or movies that Harry Potter would use a microwave to teach bread). Nevertheless, “teach” could still be as easily processed as real-world plausible information in the late stage of fictional comprehension. These findings suggested that comprehenders are able to accommodate world knowledge violations even without specific contextual support (although this accommodation requires additional time and is thus relatively delayed in real-time processing).

The discrepancy between the results of these studies may be due to the different stimuli they used. Research has shown that in contexts involving unfamiliar fictional characters, simply alerting comprehenders to the fantasy world is not sufficient (Experiments 2a and 2b, Creer et al., 2018); rather, elaboration on the fictional setting is necessary for comprehenders to overcome processing difficulties caused by real-world implausible information (Experiment 1, Creer et al., 2018). Therefore, the absence of a reversed world knowledge effect in Cook and Myers (2004) may be attributed to the lack of sufficient elaboration on the fictional setting, which was presented in just one simple sentence. In contrast, familiar fictional characters are closely associated with supernatural actions, and this association can be readily and rapidly accessed from comprehenders’ long-term memory, making contextual elaboration less necessary (Filik & Leuthold, 2013; Warren et al., 2008).

### Real-world versus contextual knowledge in relatively unconstraining scenarios

Unlike the stimuli used in most previous studies, in many real-life situations, people often discuss real-world implausible events without involving familiar fictional characters and/or providing an extensive discourse context or detailed set-up story. For example, a conversation might begin with a sentence like “I am reading an interesting book about a boy *travelling to the past*”. In this case, comprehenders may process the real-world implausible “travelling to the past” effortlessly, even though there are no explicit cues biased towards such information (since “a boy *travelling to London*” would be just as acceptable in this context). Therefore, in order to develop a more comprehensive understanding of how real-world implausible information is processed in everyday conversations, it is crucial to focus on these relatively “loose” or “unconstraining” contexts, which do not have strong indications towards a specific consequence but allow for a variety of possibilities that can be either real-world plausible or implausible.

Common examples of relatively unconstraining scenarios include children’s games and video games, where role-playing can feasibly involve both real-world (e.g., doctors and patients) and unreal (e.g., superheroes and monsters) roles; novels, movies, and TV series, where the protagonist can conduct both real-world plausible (e.g., travelling to London) and implausible (e.g., travelling to the past) actions; dreams, fantasies, and wishes, which can entail both practical (e.g., winning the lottery) and imaginary (e.g., having wings) events; and curses, which can involve both real-world possible (e.g., hoping someone fails an exam) and impossible (e.g., hoping someone turns into an ugly toad) events, to name but a few. This type of context serves as a useful testbed to explore, in a more fine-grained way, the level of contextual constraints required for real-world implausible information to be favoured over more plausible counterparts.

In light of this, an important question arises: in this type of relatively unconstraining scenarios, does real-world and contextual knowledge still affect language comprehension in the same way as in contexts with explicit constraints? To be more specific, will comprehenders still prefer real-world implausible information even when there is no strong bias towards it, or will comprehension mainly be driven by real-world knowledge instead?

Another important issue for investigation is the cognitive mechanisms underlying the comprehension of unrealistic contexts that are relatively unconstraining. As discussed earlier, the contextual effects observed in existing studies were mainly a result of reduced processing difficulties for real-world implausible information, and this reduction was attributed to the explicit and specific contextual support for such implausible information. Whether and how this pattern would change when there is no explicit bias towards either real-world plausible or implausible information is currently unknown. Specifically, if world knowledge has a greater impact on comprehension than contextual cues, as in realistic contexts, does this mean that context has no effect at all, or that context still affects comprehension, just not as strongly as real-world knowledge? If contextual cues have a greater impact on comprehension than world knowledge, which factors contribute to the diminishing or reversing of the world knowledge effect? Is it a result of increased processing efforts for real-world plausible information, or does it stem from decreased processing efforts for real-world implausible information as has been found in many previous studies? Answering these questions requires a comparison between the comprehension of an unconstraining unrealistic context and a realistic baseline condition, a comparison that has been absent in existing studies (e.g., Rohde et al., 2021).

### Theoretical and methodological issues

Existing theories seem to indicate opposing answers to these questions. On the one hand, real-world knowledge undoubtedly has a significant impact on language comprehension, especially in unconstraining scenarios where there is no strong competition from the context. According to the Resonance-Integration-Validation (RI-Val) Model, both general world knowledge and contextual information are continuously activated during language processing, but which one of them “wins” is determined by the strength of their memory trace and their featural overlap with the incoming word (Cook & O’Brien, 2014; O’Brien & Cook, 2016a, 2016b). With regard to memory trace, in contexts where the unrealistic setting is not elaborated or causally connected (e.g., “In Mary’s dream”), its memory trace is relatively weak. In contrast, world knowledge is stored in long-term memory, and thus can be “strongly encoded, elaborated, and interconnected” with the upcoming content (O’Brien & Cook, 2016a, p. 256). Consequently, the memory trace of world knowledge is likely to be stronger than that of the context. Hence, world knowledge may have a stronger impact than context in such cases. More importantly, with regard to featural overlaps, there is little information that shares a sufficient overlap with the context for it to be activated in comprehenders’ working memory. For example, in the sentence “In Mary’s dream, she put some fresh meat in the *refrigerator/wardrobe”*, neither the real-world plausible “refrigerator” nor the real-world implausible “wardrobe” is directly connected to the “dream” context in any meaningful way, so neither of these words would be activated through context. However, since “refrigerator” is associated with “the place to put fresh meat” through comprehenders’ real-world knowledge, “refrigerator” might still be activated while “wardrobe” might not. In other words, even if comprehenders might predict something real-world implausible in such a context, it seems extremely difficult for such information to be activated. Then, it follows that world knowledge should have a stronger impact than contextual cues on the comprehension of such unrealistic scenarios that are relatively unconstraining.

On the other hand, even without explicit cues towards world knowledge violations, an unrealistic context itself may, to some extent, be enough to license a “willing suspension of disbelief’’ (Coleridge, 1817/2009) that guides comprehenders to apply a more lenient interpretation strategy (Huang & Gordon, 2011). As pointed out by the dynamic generative framework (Kuperberg, 2016), when a context fails to provide enough evidence for comprehenders to form specific expectations for the upcoming information, they will, instead, use multiple types of information within the context to construct the entire situation model in a more general way. This leads them to make predictions for the coarse-grained semantic properties of the incoming words based on the constructed situation model, and any word that matches these properties can be preferred, even if they are lexically unexpected or implausible. “Dreams” can be closely associated with events that are unusual in real life (i.e., events with a relatively low level of real-world plausibility). Therefore, when processing “In Mary’s dream, she put some fresh meat in the …”, although comprehenders lack sufficient cues to form a specific expectation of the next word, they will be able to infer that the described event may not be something highly plausible in the real world. Therefore, they might prefer the real-world implausible “wardrobe” over the real-world plausible “refrigerator”, which means that the comprehension of such relatively unconstraining unrealistic scenarios is still mainly driven by context.

Importantly, as pointed out by Colvin and Warren (2020), these two models are not in opposition but rather complementary. Specifically, the generative framework focuses more on the top-down aspect of comprehension, that is, comprehenders use their knowledge and expectations at higher levels (e.g., the event level) to guide the interpretation of linguistic input at lower levels (e.g., the lexical level). On the other hand, the RI-Val model mainly emphasises the bottom-up aspect of comprehension, that is, comprehension starts from low-level features (e.g., the shared features between incoming contents and information in comprehenders’ active memory) and gradually builds up to higher-level understanding. Since these two aspects predict two opposite pathways for comprehension in relatively unconstraining scenarios, investigating this issue would also help provide some interesting insights into the interplay between the top-down and bottom-up aspects of language comprehension.

Empirical evidence has also been limited regarding the comprehension of relatively unconstraining scenarios. In an ERP experiment (Călinescu et al., 2020), participants were required to read realistic and unrealistic clauses that contained either real-world plausible or implausible information (e.g., “Magnus knows_realistic_/dreams_unrealistic_ that mosquitos live off blood_plausible_/vodka_implausible_”), and results showed that implausible information evoked a larger N400 even in the “dream” condition, indicating the presence of the world knowledge effect in an unrealistic context that is relatively unconstraining. However, the observed N400 effect only reflected the immediate processing of the critical word, while the contextual effect might be relatively delayed and thus only observable in later parts of the scenario (e.g., Rohde et al., 2021; Warren et al., 2008). Therefore, it is important to conduct an offline experiment or to include a spillover region after the critical word in an online experiment, so that we can examine in more detail the interplay between real-world knowledge and context at later stages of language comprehension.

In another self-paced reading experiment (Experiment 3a, Rohde et al., 2021), participants read a real-world context which implied that something unusual was about to be mentioned (e.g., “I wanted to tell you what I saw” in a text-message dialogue), followed by information that was either real-world plausible or implausible (e.g., “the price for the socks was actually $2_plausible_/$150_implausible_, that’s what I saw”). Results showed a reversed world knowledge effect at the sentence-final region — the reading times were longer for the plausible information than their implausible counterparts, suggesting that comprehenders do not necessarily need specific cues to predict something real-world implausible. However, this effect disappeared when the word “actually” was removed from the context (Experiment 3b, Rohde et al., 2021). Previous studies have shown that “actually” is closely associated with counter-expectations (e.g., Rohde et al., 2016). Therefore, it seems that the reversed world knowledge effect observed was caused by the use of “actually” rather than the relatively unconstraining scenarios themselves. Besides, since their experimental design did not contain a baseline condition (i.e., a factual condition that can only accommodate real-world plausible information), it remains unclear whether the reversed world knowledge effect was caused by increased processing efforts for real-world plausible information or decreased processing efforts for real-world implausible information.

## The current study

The current study aimed to address these issues by using dream scenarios. One distinct feature of dreams is that there are no constraints on whether and how the content of a dream differs from the real world. Dreams may, to some extent, reflect certain aspects of one’s feelings and experiences in real life, so it is perfectly normal for people to dream about scenarios identical to the real world; but at the same time, it is also common for dreams to feature real-world implausible scenarios. Furthermore, since in most cases, people choose to talk about their dream only when something interesting (maybe less expected) is involved, comprehenders may actually expect such information to be mentioned in a dream scenario. Thus, the use of dream scenarios makes it possible to observe the effects of contextual knowledge on promoting expectations for real-world implausible information, even in weakly constraining contexts. Nevertheless, due to the lack of specific and explicit contextual support for such implausible contents, they are often less predictable and may thus lead to comprehension difficulties. As a result, dream scenarios can serve as a good testbed for examining the interplay between real-world and contextual knowledge.

Importantly, while there are many real-world examples of relatively unconstraining contexts (e.g., games, novels, movies, etc.), constructing a realistic ‘baseline’ condition is extremely challenging for most of them. Consider the sentence “I watched an interesting movie about …”. In order to create a baseline condition that is experimentally comparable, we need to find a word to replace “movie” that only allows real-world plausible information to be mentioned in the following text. Such replacements are hard to find, as most things that people watch often include a blend of realistic and fantastical elements. The few exceptions, such as “news broadcast”, have multiple constraints on the content that can follow (e.g., only certain topics are typically covered in the news). However, for dream scenarios, it is (at least somewhat) easier to create corresponding realistic scenarios that are experimentally comparable (e.g., “I had an interesting day vs. dream yesterday.”), which is crucial for investigating the cognitive mechanisms underlying the comprehension of relatively unconstraining contexts.

In summary, the current study focuses on relatively unconstraining scenarios, a type of scenario commonly involved in real-life communication but often overlooked in existing research. We believe that the results of the present study could advance current theories of language comprehension in several significant ways. Firstly, since most existing studies used specific contextual constraints to accommodate real-world implausible content, our investigation into relatively unconstraining scenarios can provide language researchers with critical insights into how much contextual support is needed for comprehenders to favour real-world implausible information over plausible information during incremental processing. Secondly, the current study sheds light on the cognitive mechanisms underlying the interplay between real-world and contextual information in relatively unconstraining contexts, an important question left open by existing studies – for example, if an attenuated or reversed world knowledge effect is found in dream scenarios, is this effect due to increased processing efforts for real-world plausible information or decreased processing efforts for real-world implausible information (compared with realistic scenarios)? Furthermore, as discussed in *Theoretical and methodological issues*, since top-down and bottom-up processing indicates different mechanisms underlying the comprehension of dream scenarios, the current study can offer valuable insights into the relative contributions and availability of these processing mechanisms during language comprehension. This could significantly enhance our understanding of how comprehenders navigate and integrate various types of information in real time, especially in contexts that do not provide clear constraints.

## Experiment 1: Sentence completion task

This experiment used a sentence-completion task to investigate whether comprehenders’ expectations are primarily driven by world knowledge or context in an unrealistic scenario that is relatively unconstraining. To this end, we compared the real-world plausibility and variability of completions in unrealistic scenarios with those in realistic scenarios. Specifically, the experimental stimuli portrayed a protagonist sharing with others either their real-life experiences or the content of their dreams, which rendered the scenario either realistic or unrealistic. Then, we described this experience or dream, in which the protagonist performed a certain action, and we asked the participants to complete the scenario by describing how this action was performed by the protagonist.

The unrealistic setting was constructed by the description of “dreams”, considering that the content of a dream is hardly constrained by real-world knowledge, and this setting could thus facilitate the comprehension of both real-world plausible and implausible information. As discussed in the *Introduction* section, most previous studies adopted strongly biased contextual cues to indicate world knowledge contradictions. In the context of a dream, however, both real-world plausible and implausible events seem highly acceptable. Therefore, participants were not guided by any explicit contextual constraints during the experiment, which allowed a more fine-grained inspection of the interaction between real-world and contextual knowledge.

Notably, comprehenders’ expectations in unrealistic contexts may be affected by context length. It has been found that a longer context could motivate comprehenders to construct a situation model of the discourse and to engage in deep comprehension (Brothers et al., 2020). Under this circumstance, comprehenders are heavily focused on contextual information, so the impact of world knowledge is likely to be eliminated in longer contexts, even in situations where there is no strong competition from context (Walsh et al., 2018). Besides, if comprehenders do expect something real-world implausible, this expectation may grow stronger after they have read a longer context which, as yet, contains no such information. However, it is difficult to decide how long a context should be to be defined as “short” or “long”. To address this problem, we included context length as a continuous variable using the number of words before the critical word in the description of one’s experiences or dreams. The range of variation in context length was determined based on existing works investigating the role of context length in language processing (e.g., 4–9 words, Chang et al., 2020; 7–17 words, Yang et al., 2015; 8–16 words, Yang et al., 2018). In the current experiment, the lower limit of the range was similar to that of the previous studies (four words), and the upper limit was a lot higher than that of the previous studies (29 words) to increase the chance of detecting the possible effects of a “long context”.

If real-world knowledge has a greater impact than contextual cues in relatively unconstraining unrealistic scenarios, participants should provide real-world plausible completions in both realistic and unrealistic scenarios. Presumably, there should be no difference between the real-world plausibility (assessed independently of the realistic/unrealistic setting) and variability of completions in these two types of scenarios. By contrast, if contextual cues have a greater impact than world knowledge in unconstraining unrealistic scenarios, although comprehenders would not have specific predictions, they would prefer relatively less plausible information in general. In particular, this effect might be modulated by the length of context. If this is the case, the completions provided in unrealistic scenarios should be open to a variety of possibilities instead of being limited to high real-world plausibility options as in realistic scenarios. Hence, the completions in the unrealistic scenarios should have lower real-world plausibility and higher variability than those in the realistic scenarios, and these differences might increase with the context becoming longer.

### Method

#### Participants

Fifty-five participants were recruited. We excluded data from three participants due to their failure to pass any of the attention checks, leaving 52 participants in the final data analysis (28 females, one non-binary; mean_age_ = 20.4 years; *SD*_age_ = 1.57; age range = 18–23 years). The final 52 participants were all native speakers of English with no history of neurological or psychiatric diagnoses, and they were all British citizens. Participants were recruited via Prolific (www.prolific.co), and were compensated with payment. All materials, data and analysis code are available via the Open Science Framework at: https://osf.io/de6gc/.

#### Design and materials

As illustrated in Table 1, this experiment had two conditions: realistic versus unrealistic contexts. *Context length* was also included as a continuous between-item factor in the data analysis.

**Table 1:** Experimental conditions and exemplar stimuli in Experiment 1

Conditions	Examples
Realistic	Mary is telling her friend what she did on Sunday. That day, she drove to the nearest grocery store with her husband, bought some fresh meat and vegetables, and then put them in ____.
Unrealistic	Mary is telling her friend what she dreamt on Sunday. In her dream, she drove to the nearest grocery store with her husband, bought some fresh meat and vegetables, and then put them in ____.

Each item contained two parts. The first part was an initial sentence setting up the background, which framed the scenario as realistic or unrealistic (e.g., what Mary *did* vs. what Mary *dreamt*). Since the description of dreams usually appears in a dialogue, we indicated that the protagonist was having a conversation with another person in the opening sentence (e.g., “Mary is telling her friend what she *did/dreamt* on Sunday”). The second part consisted of several clauses, describing a realistic or unrealistic event that ended with a prepositional phrase (e.g., “In her dream, she drove to the nearest grocery store with her husband, bought some fresh meat and vegetables, and then put them in ____”). The prepositional phrase had a “preposition + noun phrase” structure, and the noun phrase was truncated. Participants were instructed to complete the scenario with a noun phrase that they thought could best fit in the context.

We counted the number of words before the missing part in the description of one’s experiences or dreams (e.g., “she drove to … put them in”), and used it as the length of context variable in the data analysis (mean = 15.67 words, *SD* = 8.40, range = 4–29 words). Context length was treated as a between-item factor, as it remained the same in different conditions of an item, but varied across different items.

Twenty-four sets of critical scenarios were used in the experiment. Two lists were created using a Latin square procedure, with each list containing 24 critical stimuli (12 per condition) as well as 26 filler stimuli. The 26 filler stimuli also varied in length, but they were all realistic and did not contain any implausible information. The order of all the items was randomized, and participants were randomly assigned to the testing lists.

#### Procedure

The experiment was created and administered through Microsoft Forms, a web-based survey tool. First, participants were asked to give their consent to participate in the experiment and to provide their demographic information. Then, they were instructed to read each sentence attentively and complete the sentence with a noun phrase that they thought would best fit in the context. There were no other restrictions on what they filled in. Although there was no time limit for the experiment, participants were encouraged to complete each scenario as quickly as possible without overthinking their decisions.

To ensure participants’ attentiveness during the experiment, we also included five attention checks which instructed participants to fill in a given word that would lead to severe semantic or syntactic violations (e.g., “Caroline is enjoying a nice vacation in Paris. She has been looking forward to this trip for a long time. This is an attention check. Please fill in ‘one’. On her first day in Paris, she went to Disneyland with ___”).

#### Data analysis

Data were analysed using R, Version 4.1.3 (R Core Team, 2022). In order to compare the real-world plausibility of completions in realistic and unrealistic scenarios, we removed the realistic/unrealistic setting from all the items, and then asked two raters to judge the real-world plausibility of participants’ completions in the scenario (e.g., “Mary drove to the nearest grocery store with her husband, bought some fresh meat and vegetables, and then put them in *the refrigerator*”) using a 7-point scale (1 = totally implausible; 7 = highly plausible). The two raters were both native English speakers and British citizens. They did not participate in any of the main experiments, and were blind to the aims of the study. Cohen’s kappa was computed to assess the interrater reliability, which indicated an almost perfect agreement between the two raters (based on the criteria proposed by Landis & Koch, 1977), *k* = 0.96, *Z* = 33.90, *p* < .001. For items with differing ratings between the two raters (n = 93, or 15.46% of all items), a final rating was reached through discussion.

Then, we analysed the ratings by adopting linear mixed-effects models (Baayen et al., 2008) using the *lme4* package (Bates et al., 2015), which included variance associated with each individual participant and each experimental item instead of simply averaging their contributions. In the model, fixed effects included *context* (realistic vs. unrealistic, coded as − 0.5 vs. 0.5) and the z-scored values of *context length* (continuous); random effects included *participant* and *item*.

To fit the best model, we started with the model containing a maximal random effects structure by including the interaction term as by-subject and by-item random slopes (Barr et al., 2013), that is, context * Z_context length + (context * Z_context length | subject) + (context | item). Context length was excluded from the by-item random slope because it was a between-item factor. Then, the model was trimmed stepwise to facilitate model convergence, by first removing the interaction and then the simple effect as by-subject and/or by-item random slopes based on the amount of variance it explained in the non-converging model. The largest converged model was adopted as the final model. The significance of the effects in the final model was determined via type III ANOVA with Satterthwaite’s method of approximating degrees of freedom implanted in the *lmerTest* package (Kuznetsov et al., 2017).

In addition, for each item, we also calculated the entropy for the set of completions in the realistic and unrealistic conditions separately. Generally speaking, entropy provides a measure of the amount of information needed to accurately describe and represent a given event. Thus, it can be used to quantify the variability within a set of contents – for a set of contents with greater variability, more information is needed to represent it, leading to higher entropy scores. Consequently, higher entropy scores indicate greater variability and vice versa. Moreover, since participants are likely to generate more varied completions in less constraining contexts, entropy can, in a way, serve as an indicator of how constraining the context is, providing additional insights about our stimuli.

Entropy scores were calculated using the entropy package (Hausser & Strimmer, 2021), and all the data were manually coded prior to the calculation. We treated the same noun with different articles (i.e., a/the) and various possessive adjectives (e.g., his/her/their) as identical responses. However, if other adjectives were used to describe the noun, we coded them as a different response. For example, “a/the/his/her kitchen” was coded as “A”; “a new kitchen” was coded as “B”; “a/the/his/her bedroom” was coded as “C”. Considering the small number of data points (altogether 48 data points, i.e., for each of the 24 items, an entropy score could be computed for the set of completions in both the realistic and unrealistic conditions, yielding 24 × 2 entropy scores), we conducted a permutation-based ANOVA test using the *permuco* package (Frossard & Renaud, 2021). The fixed effects included *context* (realistic vs. unrealistic, coded as − 0.5 vs. 0.5) and *context length* (continuous). For each permutation, we randomly shuffled the data within each item and then recalculated the difference in the entropy between the realistic and unrealistic conditions. The final results were calculated based on 100,000 permutations to ensure robustness and reliability.

### Results

**Fig. 1:**
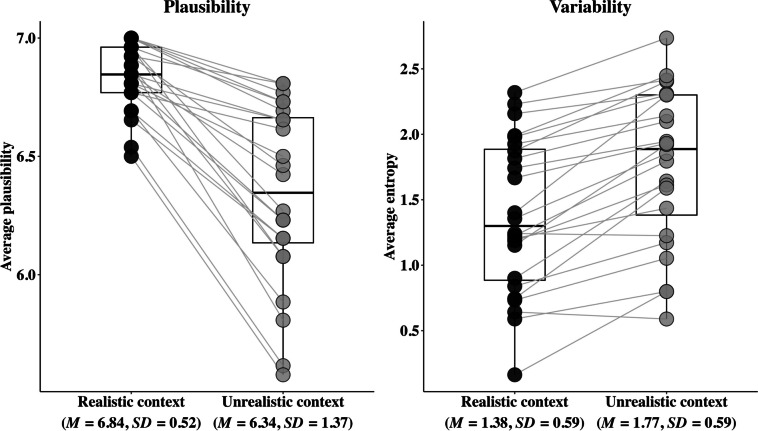
Experiment 1 real-world plausibility and variability of completions by item and by condition. Each point represents an individual item, and the grey lines connect the same item under two different conditions

Figure 1 visualizes the real-world plausibility and variability of completions for each item in realistic and unrealistic contexts.

For real-world plausibility, the analysis revealed only a significant main effect of context, *β* = −0.50, *SE* = 0.05, *t* = −9.13, *p* < .001, indicating that the plausibility of completions in the realistic context (mean = 6.83) was significantly higher than that in the unrealistic context (mean = 6.34). Particularly, more completions were rated as highly real-world plausible (6 or 7) in the realistic context (*n* = 600, or 96.15% of all completions) than in the unrealistic context (*n* = 528, 84.62%); but fewer completions were rated as highly real-world implausible (one or two) in the realistic context (*n* = 1, 0.16%) than in the unrealistic context (*n* = 30, 4.81%). Neither the main effect of context length nor the interaction between context and context length was significant (*p*s > .1).

For variability, the analysis also revealed a significant main effect of context, *F* (1, 22) = 56.98, *p* < .001, indicating that the entropy of completions in the unrealistic context (mean = 1.77 bits) was significantly higher than that in the realistic context (mean = 1.38 bits). Again, no effects of context length were significant (*p*s > .1).

### Discussion

Experiment 1 aimed to explore whether and how comprehenders’ expectations may be modulated by an unrealistic scenario that is relatively unconstraining. Specifically, we investigated this question by examining what information participants chose to mention when completing an unrealistic (vs. realistic) context. The results revealed that in unrealistic contexts, participants’ completions exhibited lower real-world plausibility and higher variability compared to those in realistic contexts.

These results clearly demonstrated that participants expected something unusual (relatively less plausible) in the unrealistic contexts, indicating significant influences from context even without the presence of explicit constraints. Expectations in realistic contexts are often based on the likelihood of the content appearing in the context, which is assessed by comprehenders’ real-world knowledge. Since only a limited number of completions may be considered as highly plausible in a certain context, participants’ completions were similar to each other, resulting in relatively lower entropy. By contrast, in an unconstraining unrealistic context, participants preferred relatively less plausible information in general. Since different individuals are likely to think of different completions that they perceive as less plausible in a given context, the completions were more varied in unrealistic contexts.

Interestingly, although completions were relatively less plausible in unrealistic contexts, a majority of them (84.62%) still exhibited a high level of real-world plausibility (ratings = 6 or 7). Therefore, it seems that in an unrealistic scenario that is relatively unconstraining, while context does have a significant effect on expectations, it does not completely override real-world knowledge, which is contrary to the findings of many previous studies (e.g., Nieuwland & Van Berkum, 2006). Rather, comprehenders’ expectations are affected by both real-world and contextual knowledge simultaneously. We return to this point in the *General discussion*.

Of note, there were also some highly implausible completions in unrealistic contexts (i.e., completions whose plausibility ratings were 1 or 2, *n* = 30), e.g., “she went surfing in *space*”, “he went to sing karaoke in *the lake*”, “she washed her hair in *ink*”, “she made a pizza and then put it in *her mouth hole [sic], and her mouth hinged open like a wheelie bin lid*”, etc. These results clearly confirmed that comprehenders’ expectations can be affected by contextual cues even without the use of explicit constraints. Thus, it is crucial to compare, in a more direct way, how real-world plausible and implausible information is comprehended in relatively unconstraining scenarios during online processing. To this end, we conducted Experiment 2, which investigated the time course of how participants processed implausible (vs. plausible) information in a realistic or unrealistic scenario.

## Experiment 2: Self-paced reading task

The results of Experiment 1 showed that while completions were more varied in unrealistic contexts than in realistic contexts, most of them were still highly plausible. This seems to suggest that comprehenders considered real-world and contextual knowledge simultaneously in an unrealistic context. However, it remains unclear when and to what extent each knowledge type influences incremental processing. To answer this question, Experiment 2 used a self-paced reading task to further explore the detailed time course of the competition between real-world and contextual knowledge in an unconstraining unrealistic scenario. To this end, we compared the reading times for real-world plausible versus implausible information in unrealistic contexts with those in realistic contexts.

Table 2 contains an example of the experimental items, which were presented word-by-word. If language comprehension is predominantly constrained by real-world knowledge in a relatively unconstraining unrealistic scenario, processing real-world implausible information should require extra cognitive effort and resources. Thus, we predicted longer reading times for the implausible condition than the plausible condition in both realistic and unrealistic contexts. This effect may be observed in the critical (e.g., *wardrobe*/*refrigerator*) and/or spillover regions (e.g., *after*, *she*, *went*, *back*, and *home*; e.g., Cook & Myers, 2004; Walsh et al., 2018).

**Table 2:** Experimental conditions and exemplar stimuli in Experiment 2

Conditions	Examples
Realistic-plausible	Mary is telling her friend what she did on Sunday. That day, she drove to the nearest grocery store with her husband, bought some fresh meat and vegetables, and then put them in the *refrigerator* after she went back home.
Realistic-implausible	Mary is telling her friend what she did on Sunday. That day, she drove to the nearest grocery store with her husband, bought some fresh meat and vegetables, and then put them in the *wardrobe* after she went back home.
Unrealistic-plausible	Mary is telling her friend what she dreamt on Sunday. In her dream, she drove to the nearest grocery store with her husband, bought some fresh meat and vegetables, and then put them in the *refrigerator* after she went back home.
Unrealistic-implausible	Mary is telling her friend what she dreamt on Sunday. In her dream, she drove to the nearest grocery store with her husband, bought some fresh meat and vegetables, and then put them in the *wardrobe* after she went back home.

In contrast, if real-world knowledge only has a weak influence on language comprehension in a relatively unconstraining unrealistic scenario, the world knowledge effect may only be observable, if observable at all, during the initial stage of language comprehension, with the contextual effect becoming stronger at later stages (e.g., Filik & Leuthold, 2013; Warren et al., 2008). Moreover, as comprehenders integrate the semantic propositions into the wider context at the end of the scenario (i.e., the “wrap-up effect”, which is typically detected at the final region of a sentence), they would focus more on the fact that the described event occurs within a dream scenario, and thus form stronger expectations for less plausible information at this stage (Rohde et al., 2021). Following this hypothesis, in realistic contexts, we might see a world knowledge effect, evidenced by longer reading times for the implausible condition compared with the plausible condition, in both critical and spillover regions. In unrealistic contexts, however, we might only observe this effect at the initial stage, that is, the critical region (e.g., *wardrobe/refrigerator*) and/or the initial spillover regions (e.g., *after*). Then, it might be attenuated or even reversed towards the end of the scenario, that is, the last few, particularly the final, spillover regions (e.g., *home*). Furthermore, the world knowledge effect may also be modulated by the length of the context, as the impact of world knowledge may be eliminated in longer contexts (Walsh et al., 2018). If this is the case, we predicted an interaction between real-world plausibility and context length in the unrealistic scenarios – the size of the world knowledge effect should decrease with the context becoming longer.

One potential issue with this experimental design is that repeated exposure to implausible information could gradually undermine participants’ sensitivity to plausibility, potentially altering their perception of what qualified as implausible (Rohde et al., 2021). It has been found that language comprehension is affected by the broader experimental context. For example, in an ERP experiment conducted by Brothers and colleagues (2017), in the entire set of scenarios (180 filler items alongside 60 critical items), when a large portion of them (the 180 fillers) ended with highly expected words, expected words were read faster than unexpected words in the 60 critical scenarios. However, this effect disappeared when most scenarios (the 180 fillers) had unexpected endings. For the current experiment, even though participants may not expect implausible information at first, if they keep reading scenarios containing implausible information, they might notice this pattern and start to expect its recurrence in the upcoming scenarios. Consequently, the results may not be a reliable reflection of participants’ typical reactions during discourse comprehension. In order to address this issue, we used a large number of filler items (2.5 times the number of critical items) to encourage participants to read the experimental materials as naturally as possible without forming certain expectations about the content of upcoming scenarios. Since only half of the critical items seen by each participant contained implausible information, such items only took up 14.3% of all the items seen by each participant, which was a lot lower than existing studies (e.g., 50%, Kravtchenko & Demberg, 2015; 30%, Kravtchenko & Demberg, 2022; 33.3%, Rohde et al., 2021).

### Methods

#### Participants

One hundred and twenty-eight participants were recruited from the University of Nottingham. The data from one participant was lost due to technical issues with the experimental platform. We also excluded data from 24 participants due to low accuracy in the comprehension tasks (below 80%, *n* = 14) or excessive outliers (more than 30%, *n* = 10, see *Data analysis* for the detailed criteria used to exclude outliers), leaving 104 participants in the final data analysis (91 females; mean_age_ = 18.87 years; *SD*_age_ = 0.68; age range = 18–21 years). This sample size (n = 104) exceeds the sample size used in published self-paced reading studies on how world knowledge and context influence language comprehension that adopted a 2 × 2 factorial design and used about 24 critical items (e.g., *n* = 40, Williams et al., 2018; *n* = 40, Creer et al., 2018; *n* = 50, Saerys-Foy et al., 2022). We also conducted a simulation-based power analysis using the function *R2power* in the R package *mixedpower* (Kumle et al., 2021) based on the data of a previous relevant study (Saerys-Foy et al., 2022). The simulation analysis showed that 104 participants were needed to reach 90% power.

The final 104 participants were all native speakers of English with no history of neurological or psychiatric diagnoses, and they were all British citizens. Participants were recruited through the SONA system, a research participation scheme for undergraduate students, and were compensated with course credits.

#### Design and materials

As illustrated in Table 2, this experiment applied a 2 × 2 factorial design, with *context* (realistic vs. unrealistic) and *real-world plausibility* (plausible vs. implausible) as the two within-subject factors. *Context length* was also included as a continuous between-item factor in the data analysis.

The materials were adapted from Experiment 1. Each item contained three parts. The first part set up a realistic or unrealistic background, which was the same as Experiment 1. The second part used several clauses to describe the realistic or unrealistic event that always ended with the critical word, which was either real-world plausible (e.g., “putting meat and vegetables in the *refrigerator*”) or implausible (e.g., “putting meat and vegetables in the *wardrobe*”). The third part was a five-word spillover phrase (e.g., “before she went back home”), which was always identical across all conditions of the same item, and was always in the same sentence as the critical word with no punctuation in between. This means that the Spill5 region was always the last word of the sentence. Table 3 presents the means and standard deviations of word length in the six regions of interest (one critical and five spillover regions).

**Table 3:** Experiment 2 mean and standard deviation (SD) of word length in regions of interest

Regions	Critical	Spill1	Spill2	Spill3	Spill4	Spill5
mean_length_	6.46 letters	5.33 letters	3.00 letters	3.71 letters	2.71 letters	4.96 letters
*SD*_length_	2.17	0.92	0.78	1.49	0.81	1.52

In order to manipulate the plausibility of the critical words, the plausible information aligned with real-world knowledge, and might be expected based on the context or the action itself. For example, a *refrigerator* is a common place for people to store their meat and vegetables. On the other hand, the implausible information was not predictable at all, but was physically possible to be used for the action. For example, we can actually put meat and vegetables in the *wardrobe*, although normally we would not. However, since the plausible and implausible words may differ in many aspects (e.g., word length, frequency, etc.), it may not be ideal to compare them directly. To address this issue, the materials were counterbalanced such that, across participants, each critical word (e.g., *refrigerator*) appeared as both plausible (e.g., putting meat and vegetables in the *refrigerator*) and implausible (e.g., hanging clothes in the *refrigerator*) in two different contexts, respectively, but the same word was only encountered once by the same participant.

Again, we counted the number of words before the critical word in the description of one’s experiences or dreams (e.g., “she drove to … put them in the”), and used it as the length of context in data analysis (mean = 16.67 words, *SD* = 8.40, range = 5–30 words). Since the article before the critical world (a, an, the) was also included in the context length, the context lengths in Experiment 2 were one word longer than those in Experiment 1.

To test the validity and reliability of the stimuli, we conducted two separate pre-tests, including one cloze probability test and one plausibility rating test. All the participants in the pre-tests were native English speakers who did not participate in the self-paced reading experiment. They were recruited via Prolific (www.prolific.co).

First, we used the results of Experiment 1 to measure the cloze probability of the critical words in different conditions. Linear mixed-effects models detected a significant interaction between context and plausibility (*β* = 0.13, *SE* = 0.03, *t* = 4.95, *p* < .001). Post hoc analysis showed that the cloze probability of plausible critical words in realistic contexts (52.72%) was higher than that in unrealistic contexts (39.74%, *β* = 0.13, *SE* = 0.02, *t* = 6.99, *p* < .001). The results indicated that the plausible critical word was less predictable in unrealistic contexts compared to realistic contexts. Since the cloze probability of the implausible critical word was zero in all items, any ease in processing implausible information we observed in the current experiment could not be attributed to cloze probability.

Then, we conducted a real-world plausibility rating test to evaluate the extent to which the events described in different conditions were perceived as real-world plausible or implausible. In this test, 30 participants were asked to judge how likely they thought a described event (e.g., “Mary drove to the nearest grocery store with her husband, bought some fresh meat and vegetables, and then put them in the refrigerator after she went back home”) was to happen in their everyday life using a 7-point scale (1 = totally unlikely to happen; 7 = highly likely to happen). For all items, the mean ratings of the implausible conditions were all between 1 and 2, and the mean ratings of the plausible conditions were all between 6 and 7, which indicated that all the plausible items had a high level of plausibility while all the implausible items had a low level of plausibility. We compared the plausibility across the two conditions using linear mixed-effects models. The results confirmed that events described in implausible conditions (mean = 1.28) were judged less likely to happen than those described in plausible conditions (mean = 6.56; *β* = −5.29, *SE* = 0.05, *t* = −98.95, *p* < .001), as intended. Since for each item, the described events under plausible and implausible conditions were identical except for the critical word, we can be confident that the observed difference in plausibility between the two conditions was caused by the critical word solely.

Twenty-four sets of critical items were used in the experiment. Four lists were created using a Latin square procedure, with each list containing 24 critical items (six per condition) as well as 60 filler items. The 60 filler stimuli had a similar structure to the critical items, and also varied in length, but they were all realistic and did not contain any implausible information. The order of all the items was pseudo-randomized, with the restriction that any two consecutive critical items were of different conditions and that at least two filler items were presented between any two consecutive critical items. Participants were randomly assigned to the testing lists.

#### Procedure

This experiment used a self-paced reading task implemented in PCIbex (https://www.pcibex.net/), a web-based tool to build and run online experiments (Zehr & Schwarz, 2018). Before the experiment, all the participants confirmed that they understood and accepted all the items in the consent form.

During the experiment, participants were instructed to read each scenario attentively and silently. Each trial began with an asterisk (*) in the middle of the screen. Participants pressed the spacebar when they were ready to read the scenarios. All the scenarios were presented word-by-word. The words initially appeared as a series of underlines obscuring the words (___ ___ ___ ___), and participants pressed the spacebar to reveal each word. The presentation was non-cumulative so previous words were replaced with underlines when the next word appeared, which means participants could only see one word at a time.

A third of the materials were followed by a statement that probed comprehension. Participants were required to judge whether the statement was true or false based on the previously read scenario by pressing one of two keys. The assignment of left/right hands to true/false responses was completely random. Reading times for each word and the accuracy of participants’ responses to the comprehension questions were recorded.

#### Data analysis

Data analysis was performed over six regions separately (one critical region and five spillover regions). Firstly, we excluded response times below 100 ms, which was too fast to read, or above 5,000 ms, which was obviously longer than required for comprehension (altogether 219 data points in the regions of interest). Then, we pruned reading times that were over 2.5 *SD*s from the grand mean (a further 261 data points in the regions of interest). All non-outlier data were included in the analysis, regardless of comprehension-question accuracy. The reading times were log-transformed to correct the rightward skew.

The data were analysed using linear mixed-effects models. In the model, fixed effects included *context* (realistic vs. unrealistic, coded as − 0.5 vs. 0.5), *real-world plausibility* (plausible vs. implausible, coded as − 0.5 vs. 0.5), and the z-scored values of context length (continuous); random effects included *participant* and *item*. We started with the model containing a maximal random effects structure, that is, context * plausibility * Z_context length + (context * plausibility * Z_context length | subject) + (context * plausibility | item). Then, we determined the final model and obtained *p* values in the same way as Experiment 1, and *p* values were adjusted using the Bonferroni correction (Benjamini & Hochberg, 1995) implanted in the *p.adjust()* function to control for multiple comparisons. For all significant interactions, post hoc analyses were conducted using the *emmeans* package (Lenth, 2023) to obtain the simple effects. The degrees of freedom in the post hoc tests were computed using the Satterthwaite approximation, and the Tukey adjustment method was adopted for the post hoc tests.

### Results

**Fig. 2:**
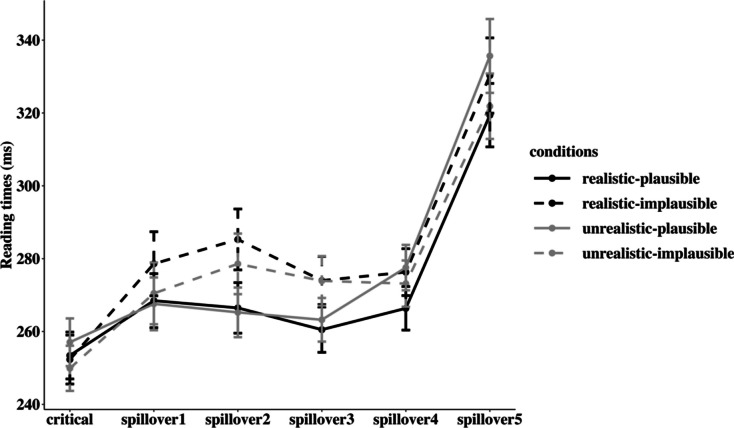
Mean reading times (ms) by condition and by region (the error bars present the 95% confidence intervals) in Experiment 2

Table 4 presents the means and standard errors of reading times by condition for the critical region and the spillover (Spill1–5) regions (visualized in Fig. 2). We then report the results from linear mixed-effects model analysis for the six regions of interest separately. The detailed statistical results are presented in Table 5.

**Table 4:** Reading times (ms) by condition and by region (means ± standard error) in Experiment 2

Conditions	Critical	Spill1	Spill2	Spill3	Spill4	Spill5
Realistic-plausible	253.42 ± 3.29	268.47 ± 3.78	266.49 ± 3.54	260.51 ± 3.18	266.37 ± 3.05	319.41 ± 4.45
Realistic-implausible	252.30 ± 3.43	278.60 ± 4.49	285.28 ± 4.27	274.00 ± 3.37	276.30 ± 3.28	330.29 ± 5.27
Unrealistic-plausible	257.05 ± 3.34	267.60 ± 3.70	265.26 ± 3.48	263.22 ± 3.06	277.56 ± 3.18	335.67 ± 5.17
Unrealistic-implausible	249.90 ± 3.16	270.47 ± 4.33	278.58 ± 4.24	273.91 ± 3.47	273.15 ± 3.24	321.85 ± 4.58

**Table 5:** Results of the linear mixed-effects models and the fixed-effects parameters in Experiment 2

Spill2 Model: context * plausibility * Z_context length + (1 + plausibility | item) + (1 | subject)					
Fixed effects	*β*	*SE*	*t*	*p*	95% CI
**(Intercept)**	**5.56**	**0.03**	**186.87**	< .001***	**[0.01, 0.07]**
context1	−0.01	0.01	−1.46	1.00	[0.22, 0.23]
**plausibility1**	**0.05**	**0.01**	**4.00**	.005**	**[5.50, 5.62]**
Z_context length	0.00	0.02	0.12	1.00	[−0.03, 0.00]
context1:plausibility1	−0.02	0.02	−1.08	1.00	[0.03, 0.07]
context1: Z_context length	0.00	0.01	−0.12	1.00	[−0.04, 0.05]
plausibility1: Z_context length	−0.01	0.01	−0.67	1.00	[−0.06, 0.02]
context1:plausibility1: Z_context length	0.03	0.02	1.59	.900	[−0.02, 0.02]
Spill3 Model: context * plausibility * Z_context length + (1 | item) + (1 | subject)					
Fixed effects	*β*	*SE*	*t*	*p*	95% CI
**(Intercept)**	**5.55**	**0.03**	**211.55**	< .001***	**[5.50, 5.60]**
context1	0.01	0.01	0.61	1.00	[−0.01, 0.02]
**plausibility1**	**0.05**	**0.01**	**5.57**	< .001***	**[0.03, 0.06]**
Z_context length	0.00	0.02	0.26	1.00	[−0.03, 0.04]
context1:plausibility1	−0.01	0.02	−0.75	1.00	[−0.05, 0.02]
context1: Z_context length	0.02	0.01	2.10	.286	[0.00, 0.03]
plausibility1: Z_context length	0.01	0.01	1.16	1.00	[−0.01, 0.03]
context1:plausibility1: Z_context length	0.03	0.02	1.54	.988	[−0.01, 0.06]
Spill4 Model: context * plausibility * Z_context length + (1 + context + plausibility | item) + (1 | subject)					
Fixed effects	*β*	*SE*	*t*	*p*	95% CI
**(Intercept)**	**5.57**	**0.03**	**211.24**	< .001***	**[0.25, 0.25]**
context1	0.01	0.01	1.14	1.00	[0.04, 0.04]
plausibility1	0.01	0.01	1.13	1.00	[−0.47, 1.00]
Z_context length	0.02	0.02	0.79	1.00	[0.00, 0.08]
**context1:plausibility1**	**−0.05**	**0.02**	**−3.28**	.008**	**[0.20, 0.21]**
context1: Z_context length	−0.01	0.01	−0.53	1.00	[5.52, 5.62]
plausibility1: Z_context length	−0.01	0.01	−0.92	1.00	[−0.01, 0.04]
context1:plausibility1: Z_context length	0.00	0.02	0.15	1.00	[−0.01, 0.03]
Spill5 Model: context * plausibility * Z_context length + (1 | item) + (1 | subject)					
Fixed effects	*β*	*SE*	*t*	*p*	95% CI
**(Intercept)**	**5.73**	**0.03**	**179.01**	< .001***	**[5.67, 5.80]**
context1	0.02	0.01	1.52	1.00	[0.00, 0.04]
plausibility1	0.00	0.01	−0.40	1.00	[−0.02, 0.02]
Z_context length	0.01	0.02	0.38	1.00	[−0.04, 0.06]
**context1:plausibility1**	**−0.07**	**0.02**	**−3.24**	.010**	**[−0.11, −0.03]**
context1: Z_context length	0.00	0.01	0.12	1.00	[−0.02, 0.02]
plausibility1: Z_context length	0.00	0.01	0.11	1.00	[−0.02, 0.02]
context1:plausibility1: Z_context length	0.00	0.02	−0.08	1.00	[−0.04, 0.04]

*** p ≤ .001; ** p ≤ .01; * p ≤ .05; + *p* ≤ .10

**Critical and Spill1 regions*.*** There were no significant effects, *p*s > .05.

**Spill2 and Spill3 regions.** A significant main effect of real-world plausibility was detected (Spill2: *β* = 0.05, *SE* = 0.01, *t* = 4.00, *p* = .005; Spill3: *β* = 0.05, *SE* = 0.01, *t* = 5.57, *p* < .001), due to longer reading times for implausible information (Spill2: mean = 282 ms; Spill3: mean = 274 ms) than plausible information (Spill2: mean = 266 ms; Spill3 = 262 ms).

**Spill4 region.** A significant interaction between context and real-world plausibility was found, *β* = −0.05, *SE* = 0.02, *t* = −3.28, *p* = .008. Post hoc analysis showed that in realistic conditions, the reading time for implausible information (mean = 276 ms) was significantly longer than that for plausible information (mean = 266 ms), *β* = −0.04, *SE* = 0.01, *t* = −2.92, *p* = .005, but this difference disappeared in the unrealistic condition, *β* = 0.02, *SE* = 0.01, *t* = 1.16, *p* = .252 (mean_plausible_ = 278 ms, mean_implausible_ = 273 ms). In addition, unrealistic contexts yielded longer reading times (mean = 278 ms) than realistic contexts (mean = 266 ms) under the plausible condition, *β* = −0.04, *SE* = 0.01, *t* = −2.80, *p* = .008, but not under the implausible condition, *β* = 0.01, *SE* = 0.01, *t* = 0.93, *p* = .360 (mean_realistic_ = 276 ms, mean_unrealistic_ = 273 ms).

**Spill5 region.** A significant interaction between context and real-world plausibility was found, *β* = −0.07, *SE* = 0.02, *t* = −3.24, *p* = .010. Post hoc analysis showed that the reading times for implausible information (mean = 330 ms) were significantly longer than for plausible information (mean = 319 ms) in realistic contexts, *β* = −0.03, *SE* = 0.01, *t* = −2.00, *p* = .046. However, this difference was reversed in unrealistic contexts – the reading times for plausible information (mean = 336 ms) were significantly longer than for implausible information (mean = 322 ms) in unrealistic contexts, *β* = 0.04, *SE* = 0.01, *t* = 2.58, *p* = .010. In addition, comparisons showed that the reading times in the unrealistic condition (mean = 336 ms) were longer than those in the realistic condition (mean = 319 ms) when the critical word was plausible, *β* = −0.05, *SE* = 0.01, *t* = −3.37, *p* < .001, whereas no difference was found when the critical word was implausible, *β* = 0.02, *SE* = 0.01, *t* = 1.22, *p* = .224 (mean_realistic_ = 330 ms, mean_unrealistic_ = 322 ms).

### Discussion

The goal of this experiment was to explore the interplay between real-world and contextual knowledge in guiding the moment-to-moment comprehension of information presented in relatively unconstraining unrealistic contexts. For this purpose, we examined how real-world implausible (vs. plausible) information was processed in a realistic or unrealistic scenario. Results showed that in realistic contexts, implausible information induced additional difficulties in language comprehension compared to plausible information, indicating the presence of the world knowledge effect. However, in unrealistic contexts, while the same world knowledge effect was also detected, it gradually diminished and ultimately reversed (though with a relatively small effect size) towards the end of the scenario.

These results seem to suggest that the impact of an unrealistic context ultimately overrides that of real-world knowledge. However, if this is the case, it follows that we should observe a reduction in the reading times for implausible information in unrealistic, compared with realistic, contexts in sentence-final regions. No such differences were found. Rather, we observed longer reading times for the plausible information in unrealistic contexts than in realistic contexts in Spill4 and Spill5 regions, which were the same regions where the world knowledge effect diminished and then reversed. This indicates that the diminishment/reversal of the world knowledge effect in the unrealistic context was mainly guided by extra processing difficulties for the plausible information, and not the other way around.

## General discussion

The experiments reported in the current paper explored the interplay between real-world and contextual knowledge within an unrealistic scenario that was relatively unconstraining. Although there were no explicit contextual constraints biased towards information with a relatively low level of real-world plausibility, participants still showed such a bias during language comprehension, signalled by an attenuated (Experiment 1) or reversed (Experiment 2, though with a relatively small effect size) world knowledge effect. Interestingly, such effects were observed in offline data (Experiment 1) and at the sentence-final regions of online data (Spill4 and Spill5 regions in Experiment 2), suggesting that real-world knowledge still guides comprehension to a greater extent than contextual information at the initial stage.

### World knowledge effect in early stages of comprehension

An important question regarding the observed contextual effect is why it was absent during the initial stages of comprehension and only emerged at the very end of the unrealistic scenarios in Experiment 2. One possible explanation could be that comprehenders’ bias towards something unusual is very weak at first, because they may assume that it is still yet to come. It is only at the end of the scenario that this bias becomes observable, as the lack of unusual information becomes apparent and there is no further content to rescue it (Rohde et al., 2021). If this is the case, we would then expect to see a reduction in the reading times for implausible information at the final regions in unrealistic, compared to realistic, contexts. However, since this difference was not significant in our analysis, this possibility does not seem very likely.

A more plausible explanation could be that, in the absence of explicit contextual constraints, comprehenders are compelled to make predictions about the upcoming content based on their real-world knowledge. For example, when reading the sentence “Mary bought some fresh meat and vegetables, and then put them in …”, comprehenders may generate a set of hypotheses regarding the likely place for Mary to put the meat and vegetables, such as “basket”, “kitchen” and “refrigerator”. Then, based on their world knowledge, they will presumably predict that “refrigerator” is the most typical option in the described situation, and thus expect “refrigerator” to be mentioned.

In an unrealistic version of this scenario (e.g., “In Mary’s dream, she …”), this process of prediction becomes extremely difficult, since the possible places for Mary to put the meat and vegetables in her dream are not specified at all. The unlimited number of potential choices obscures the way in which the upcoming content can be related to the context. Therefore, arguably, comprehenders can only generate the same set of plausible hypotheses they hold in the realistic context. While they may infer from the unrealistic setting of the context that the described event is unlikely to be highly plausible, it is very hard for them to think of information that has not been activated. As a result, they may tend to predict something that is associated with the pre-activated information (such as “kitchen” and “refrigerator”) but not highly plausible at the same time, such as “a pie” and “ice”. This would explain why most completions in Experiment 1 were plausible.

As for Experiment 2, despite the lack of unusualness of the plausible critical word (e.g., *refrigerator*), it could, at least, be easily predicted; but it was very hard for participants to predict the implausible critical word (e.g., *wardrobe*) from the information they could acquire from the context (the cloze probability of the implausible critical word was zero in all items). Therefore, it would be more difficult to integrate the unpredictable “wardrobe” into the discourse model.

### Gradually reversed world knowledge effect in later stages of comprehension

As discussed in the *Introduction*, in daily conversations, people may only choose to talk about their dreams when something interesting and newsworthy is involved. Therefore, when reading/hearing about others’ dreams, comprehenders may, in turn, expect relatively newsworthy contents to be mentioned at some point. For the current study, at the end of unrealistic scenarios, participants began to integrate the semantic propositions into the larger situation model (i.e., the “wrap-up effect”; for a review, see Stowe et al., 2018). As they reviewed and reassessed the described event as a whole, they gradually realised that the context did not contain anything unusual by mentioning the highly plausible “refrigerator”. This would be inconsistent with their prior expectation for newsworthy information, and this prediction error led to increased processing efforts for “refrigerator”, reflected in the gradual attenuation and reversal of the world knowledge effect in Experiment 2. This finding is also in line with Experiment 1, which clearly demonstrated that context, even when unconstraining, could still adjust participants’ expectations. Meanwhile, participants still found it difficult to link the implausible “wardrobe” to any information that had been activated in their working memory, so its reading time in Experiment 2 was not decreased.

To sum up, when comprehenders read an unrealistic scenario, they may expect something unusual, if not completely implausible, to be mentioned in the following text. Even when there are no available cues for comprehenders to form a specific prediction, they are still able to expect such information in a general way – that is, they “expect the unexpected”. The absence of this (un)expected information would then lead to comprehension difficulties.

Notably, although we predicted that the attenuation/reversal of the world knowledge effect would be earlier and/or stronger in longer contexts, no effects of context length were found in either experiment. This may be due to the limited amount of emphasis on the dream context. In particular, the word *dreamt*/*dream* was only mentioned twice at the very beginning of each item, and there was no other indication that the described events happened in a dream. It has been found that increasing elaboration of the unrealistic world can move up the contextual effect in time (Creer et al., 2018; Soares et al., 2023). Therefore, for the current study, it seems plausible that even without specific contextual constraints, the contextual effect may become observable in earlier stages through stronger reinforcement of the “dream” setting, or simply through more frequent mentions of the word “dream”. This would be an interesting avenue for future research.

### Real-world versus contextual knowledge during incremental processing

Our findings provide additional insights into the results of prior studies that reported a similar attenuated or reversed world knowledge effect. Most of these experiments used more constraining contexts with specific cues to support certain world knowledge violations (e.g., Creer et al., 2018; Ferguson & Sanford, 2008; Ferguson et al., 2008; Nieuwland, 2013; Nieuwland & Van Berkum, 2006). Additionally, even in the few studies that examined relatively unconstraining scenarios (e.g., Filik & Leuthold, 2013; Warren et al., 2008), familiar fictional characters (e.g., Harry Potter) were often used to accommodate real-world implausible actions (notably, in studies that had a similar design but used unfamiliar fictional characters, the attenuation/reversal of the world knowledge effect is observed only at the initial stage; see Cook & Myers, 2004). Consequently, the contextual effect observed in these studies was primarily driven by a decrease in processing difficulties for real-world implausible information.

Furthermore, although Rohde and colleagues (2021) used neither specific contextual cues nor familiar fictional characters, their experimental design lacked a baseline condition, making it difficult to determine whether the observed contextual effect was due to increased reading times for plausible information or decreased reading times for implausible information. By contrast, in the current study, the stimuli were designed so that both plausible and implausible information were supported by the context. The effects we observed were mainly guided by extra processing difficulties for real-world plausible information, but not the other way around (as there was no difference between the reading times for real-world implausible information in realistic and unrealistic scenarios).

The current study provides an important piece of the puzzle by offering additional insights into the timing at which contextual information can override real-world knowledge, a question left open by previous research. Specifically, for the contextual effect to be observable in the initial stages of comprehension, strong contextual support is necessary. Without such support, comprehenders cannot make specific predictions about implausible events and thus have to rely on their real-world knowledge (see also Filik & Leuthold, 2013; Warren et al., 2008). This suggests that the contextual effect heavily depends on explicit cues or syntactic structures (e.g., the wh-cleft structure) that specifically indicate what the implausible information will be or where it will appear. Therefore, it seems that the early stages of processing are heavily dependent on bottom-up cues.

On the other hand, in later stages of comprehension, such cues are no longer necessary for the contextual effect to be observed. Implausible information can be favoured without mentioning familiar fictional characters or providing an extensive discourse context to support such information, which suggests that comprehenders rely more on top-down mechanisms during later stages of processing. That is, comprehension difficulties can be modulated without specific constraints from the context – factors such as informativity (He & Kaiser, 2023; Rohde et al., 2021) and overall validity (Brothers et al., 2017; Lau et al., 2013) can alter comprehenders’ expectations in a general way. That being said, without these contextual supports, the comprehension of implausible information cannot be cost-free. The lack of contextual support makes it difficult for comprehenders to integrate implausible information into the discourse model, even at the end of the context. This, again, suggests the influence from bottom-up constraints. Taken together, it seems that in later stages of comprehension, both bottom-up and top-down mechanisms are working simultaneously.

### Theoretical implications

Clearly, the comprehension of information presented in relatively unconstraining contexts involves both bottom-up and top-down input from real-world and contextual knowledge, and our results can only be partially explained by either mechanism alone. Therefore, the current findings provide some interesting insights into the interplay between bottom-up and top-down processing, raising questions about many language comprehension models, such as the RI-Val Model and the dynamic framework.

The RI-Val model (Cook & O’Brien, 2014; O’Brien & Cook, 2016a, 2016b) mainly emphasises the bottom-up aspect of comprehension. It proposes that information associated with the lexical input from the scenario, either through real-world or contextual knowledge, is activated in comprehenders’ working memory. Any newly encoded content will then be linked to the activated information based on their general conceptual overlap. Finally, these linkages will be validated against all the activated information. Following this, as discussed in the *Introduction* section, in a weakly constraining scenario, plausible information can be easily validated through its shared features with previously activated contents, whereas implausible information cannot (since it is not related to either world knowledge or contextual information in any meaningful way). If this is the case, the world knowledge effect should not be attenuated/reversed even in dream scenarios, which, however, is inconsistent with our findings in the Spill4 and Spill5 regions of Experiment 2.

Nevertheless, one might argue that dream scenarios, though relatively unconstraining, may still be strong enough to override real-world knowledge during validation. Importantly, dreams themselves are not inherently associated with unusual events, as people often dream about scenarios mirroring real life. Instead, it is the act of sharing dreams with others that typically involves the mention of interesting or unexpected content. Thus, when reading conversations about dreams, comprehenders’ predictions for less plausible contents are unlikely to stem purely from bottom-up input through simple pattern-matching based on featural overlap, as suggested by the RI-Val model. Rather, such predictions require a higher-level assessment of previously read information (e.g., the event level or the discourse level).

Therefore, it seems that the current validation stage in the RI-Val model fails to fully explain the comprehension of world knowledge violations in a relatively unconstraining scenario. One potential explanation is that a fictional/counterfactual context may modulate the way comprehenders validate the information communicated by the discourse (Richter & Singer, 2018). The results of the current study suggest that such contexts provide a general indication of the event structure being communicated (i.e., top-down input), and any information that matches this event structure can be validated, even if it is unrelated to the lexical input from the context (i.e., bottom-up input). Thus, it seems plausible that top-down input may override bottom-up constraints from comprehenders’ real-world and/or contextual knowledge during validation, thus altering the criterion for validation. Alternatively, while validation was described as a purely passive, pattern-matching process in the RI-Val model, strategic processing has been widely observed in psycholinguistic research and has been incorporated into Richter’s theories of validation (e.g., Richter & Maier, 2017; Wertgen & Richter, 2023). Specifically, validation may be modulated by comprehenders’ strategic preference for a certain type of information (e.g., interesting information in dream scenarios) – mismatches with such preferences could lead to failure of validation.

Moreover, it has also been found that even in a more constraining scenario, disruptions caused by real-world/contextual knowledge violations could be accommodated by models that do not include validation (Saerys-Foy et al., 2022). Overall, the current validation stage in the RI-Val model appears to be problematic, and further research is needed to explore how validation is influenced by various factors.

The dynamic framework (Kuperberg, 2016) offers a potential alternative explanation for how comprehension may be driven by top-down mechanisms. It proposes that a comprehender seeks to infer, with as much certainty as possible, the specific event being communicated. To this end, they draw upon a generative model – a hierarchically organised set of internal representations that, at any given moment, they believe can best explain the previously encountered bottom-up input. At the top of this hierarchy lies a set of hypotheses currently held by the comprehender. These hypotheses are tested by generating probabilistic predictions, which are propagated down to lower levels of the generative model, thereby changing prior belief distributions at these levels. When new bottom-up input becomes available, the comprehender learns whether their hypotheses are supported. If not, prediction errors are propagated back up the generative model, and used to update high-level beliefs about the communicated event. Of note, if there are multiple competing cues, the more reliable cue combinations exert greater influence on the inferred event. Furthermore, in situations where contextual cues are insufficient to form a specific hypothesis about the communicated event, the comprehender instead predicts the more general event structures. These predicted event structures lead the comprehender to generate predictions for coarse-grained semantic properties of upcoming information. Content that matches these properties may then be preferred, even if it is lexically unexpected or implausible.

Following this theory, during early stages of processing in Experiment 2, “someone puts meat and vegetables in the refrigerator”, within the schema in which the protagonist just bought these items from the grocery store, is more frequently encountered and thus a more reliable prediction than “someone puts meat and vegetables in the wardrobe”. As processing continues, participants gradually realised that dream scenarios are commonly associated with unusual events. Consequently, a new generative model involving unusual events in dreams (e.g., "putting meat and vegetables in the wardrobe") became more probable than the previous generative model involving nothing unusual. Participants switched from the old model to the new one, as evidenced by the gradually reversed world knowledge effect.

However, in the later stages of processing in Experiment 2, if participants had fully adapted to the new generative model in favour of unusual events, it should have been easier for them to comprehend “wardrobe”, which was not what we observed. One possibility is that disengagement from the old model and adaptation to the new model are running in parallel. In the final regions of text, while the latter was complete, the former was not, making the influence from the old model temporarily observable. An alternative possibility is that disengagement from the old model and adaptation to the new model are part of a sequential two-stage process. In the final regions, participants were still in the middle of this switch, so both models were competing for dominance. Either way, it seems that the switch to the new model was still incomplete at the final regions of the text.

The dynamic framework posits that comprehenders modify the current generative model or switch to a new model in response to changes in their comprehension goals and/or their communicative environments. The current results suggest that two different models could be simultaneously activated in relatively unconstraining contexts, a phenomenon also observed in other types of contexts. For example, during counterfactual processing, both the factual and counterfactual representations are activated and maintained in comprehenders’ working memory (see Ferguson, 2012, for a detailed discussion). Similarly, both the realistic and fictional models are readily accessible when only a minimal fictional description is provided (Soares et al., 2023). While the dynamic framework has provided some explanation of what triggers the switch to a new model, more information is needed to understand the cognitive mechanisms underlying this process. For example, do disengagement from the old model and adaptation to the new model run in parallel or as part of a sequential two-stage process? What if the switch to the new model is incomplete before new events occur during communication? We believe that future work on language comprehension models would benefit from exploring these questions.

## Limitations

There are some limitations to the current study. While word-by-word self-paced reading is widely used and accepted in psycholinguistic research, concerns exist about its ecological validity, as word-by-word presentation may not fully reflect natural reading patterns, which could potentially influence the findings of Experiment 2. However, we observed a clear and stable world knowledge effect in realistic scenarios (across four consecutive regions) and the early stages of unrealistic scenarios (across two consecutive regions). Although this effect is relatively delayed, in general, it is still consistent with the findings of numerous previous studies using different experimental methods (e.g., ERPs, Călinescu et al., 2020; Hagoort et al., 2004; eye-tracking, Cook & Myers, 2004; Rayner et al., 2004; Warren et al., 2008), and thus seems quite reliable. This suggests that the method we used was sensitive enough to reflect online comprehension, and that our participants were actively processing the materials during the experiment. Therefore, we believe that potential influences from the word-by-word presentation method are minimal, and unlikely to undermine the validity of our results. Nonetheless, it would be interesting to replicate the current study using methods that allow participants to read in a more natural way, such as eye-tracking, to further validate the findings.

## Conclusion

Most prior work studying the interplay between real-world and contextual knowledge used specific contextual cues to indicate a strong bias towards world knowledge violations. In contrast, the current study aimed to investigate this issue in an unrealistic scenario where no such constraints are provided, a type of scenario commonly involved in real-life communications but often overlooked by existing studies. The results showed that at the early stage of comprehension, despite their preference for something unusual, comprehenders lack sufficient cues from the context to make predictions about the upcoming content, and are thus more likely to rely on their real-world knowledge. This implies that language comprehension is initially guided by world knowledge even in relatively unconstraining unrealistic scenarios. However, as the sentence progresses, the role of context gradually gains more dominance towards the end of the scenario, where comprehenders begin to assess the relationship between semantic propositions and the larger discourse context.

Our study provides additional information on how constraining a context needs to be for the contextual effect to be observable, by showing that context is powerful enough to reverse the world knowledge effect even without the use of explicit constraints (although this effect may only become observable at sentence-final regions). In particular, in the absence of specific cues, an unrealistic context is enough for comprehenders to expect real-world implausible information in a general sense, but it is not sufficient to overcome the comprehension difficulties caused by a specific instance of such information. More importantly, although there have been several studies investigating this issue, to our knowledge, our study is the first to explore the underlying mechanism driving this effect – the attenuation/reversal of the world knowledge effect was caused by increased processing efforts for real-world plausible information, not the other way around.

Our findings also indicate necessary extensions for language comprehension models, since most of them (e.g., the RI-Val model) assume that comprehension is driven by comprehenders’ preference for information that has been pre-activated by either their world knowledge or the contextual information. Rather, the current study highlights that information unrelated to both real-world and contextual knowledge in any direct way (i.e., information with extremely low cloze probability) can still be preferred in certain scenarios. Furthermore, while many language comprehension theories mention the possibility of the switch between different mental models, they fail to outline a detailed mechanism underlying this process. The current findings raise new questions about this issue, by suggesting that two conflicting models may be simultaneously activated during incremental processing.

## Data Availability

The data and materials for all experiments are available at https://osf.io/de6gc/.

## Code availability

R scripts for all experiments are available at https://osf.io/de6gc/.

## Declarations

### Open practices statement

We report all data exclusions, manipulations, and measures in the study. The research materials, data, and analysis code for all experiments are available through the Open Science Framework at: https://osf.io/de6gc/. None of the experiments was preregistered.
